# 45 Utility of the Severity-of-Illness Score for Toxic Epidermal Necrolysis (SCORTEN) in Pediatric Stevens-Johnson Syndrome Patients

**DOI:** 10.1093/jbcr/irae036.045

**Published:** 2024-04-17

**Authors:** Nina K B Gust, Rebecca M Adams, Ashley Frei, Michelle Coughlin, Justin D Klein, Elika Ridelman, Christina M Shanti

**Affiliations:** Wayne State and Children's Hospital of Michigan, Royal Oak, Michigan; Wayne State University School of Medicine and Children's Hospital of Michigan, Grosse Pointe, Michigan; Wayne State University School of Medicine, Detroit, Michigan; Wayne State University, Detroit, Michigan; Children's Hospital of Michigan, Detroit, MI; Wayne State and Children's Hospital of Michigan, Royal Oak, Michigan; Wayne State University School of Medicine and Children's Hospital of Michigan, Grosse Pointe, Michigan; Wayne State University School of Medicine, Detroit, Michigan; Wayne State University, Detroit, Michigan; Children's Hospital of Michigan, Detroit, MI; Wayne State and Children's Hospital of Michigan, Royal Oak, Michigan; Wayne State University School of Medicine and Children's Hospital of Michigan, Grosse Pointe, Michigan; Wayne State University School of Medicine, Detroit, Michigan; Wayne State University, Detroit, Michigan; Children's Hospital of Michigan, Detroit, MI; Wayne State and Children's Hospital of Michigan, Royal Oak, Michigan; Wayne State University School of Medicine and Children's Hospital of Michigan, Grosse Pointe, Michigan; Wayne State University School of Medicine, Detroit, Michigan; Wayne State University, Detroit, Michigan; Children's Hospital of Michigan, Detroit, MI; Wayne State and Children's Hospital of Michigan, Royal Oak, Michigan; Wayne State University School of Medicine and Children's Hospital of Michigan, Grosse Pointe, Michigan; Wayne State University School of Medicine, Detroit, Michigan; Wayne State University, Detroit, Michigan; Children's Hospital of Michigan, Detroit, MI; Wayne State and Children's Hospital of Michigan, Royal Oak, Michigan; Wayne State University School of Medicine and Children's Hospital of Michigan, Grosse Pointe, Michigan; Wayne State University School of Medicine, Detroit, Michigan; Wayne State University, Detroit, Michigan; Children's Hospital of Michigan, Detroit, MI; Wayne State and Children's Hospital of Michigan, Royal Oak, Michigan; Wayne State University School of Medicine and Children's Hospital of Michigan, Grosse Pointe, Michigan; Wayne State University School of Medicine, Detroit, Michigan; Wayne State University, Detroit, Michigan; Children's Hospital of Michigan, Detroit, MI

## Abstract

**Introduction:**

The Severity-of-Illness Score for Toxic Epidermal Necrolysis (SCORTEN) is a scoring system that seeks to predict in-hospital mortality for disorders affecting skin integrity. The scoring system has been validated and is widely utilized in the adult population, but not for pediatric patients. This study aims to determine the accuracy of the SCORTEN in pediatric patients.

**Methods:**

A retrospective review of pediatric patients admitted at a verified pediatric burn center with Stevens-Johnson Syndrome/Toxic Epidermal Necrolysis (SJS/TEN) from 2008 to 2022 was performed. SCORTEN risk factors were calculated.

**Results:**

Twenty-seven eligible SJS/TEN patients were identified. Three patients were excluded due to incomplete data, allowing twenty-four patients to be analyzed. Ten patients had 0-1 risk factors (3.2% mortality rate), thirteen had 2 risk factors (12.1% mortality risk) and one had 3 risk factors (35.1% mortality risk). No patients received points for age over 40, presence of malignancy, blood urea nitrogen (BUN) greater than 28 mg/dL or glucose greater than 250 mg/dL. Zero patients died during initial admission. There was no correlation between initial BUN, bicarbonate, glucose, or initial heart rate on the length of ICU stay or ventilator days. Hospital length of stay and feeding tube days were positively correlated (p < 0.001) along with length of stay and maximum TBSA (p < 0.05). Student T-tests were performed comparing the 0-1 vs. 2 risk factor groups. Those with 2 risk factors had significantly higher TBSA affected on admission and maximum TBSA recorded (32.72 ± 20.55 and 44.97 ± 24.25 vs 8.26 ± 13.55 and 16.28 ± 22.19, p = .0005 and p=0.011). Interestingly, hospital length of stay, intensive care unit (ICU) length of stay, and ventilator days were not statistically significant between those having 0-1 and 2 risk factors.

**Conclusions:**

Most SCORTEN variables (age over 40, presence of malignancy, BUN, and glucose) were not helpful in our patient population, as zero patients received points in these categories. Additionally, initial BUN, bicarbonate, glucose and heart rate showed no correlation to ICU or ventilator days. A higher SCORTEN category did not correlate to a longer hospital stay, ICU days or ventilator days. This study suggests that the SCORETEN system is not accurate in pediatric patients. A different scoring system is needed to estimate the severity of disease in pediatric SJS/TEN patients.

**Applicability of Research to Practice:**

This data suggests that SCORTEN does not have utility in a pediatric SJS population and therefore a different scoring system is necessary. In practice, it would not be valid to use SCORTEN to predict mortality or severity of disease in the pediatric SJS population as it has not been found to be accurate.

